# Abnormal obstetric shock index and associated factors among immediate postpartum women following vaginal delivery at a tertiary hospital in southwestern Uganda

**DOI:** 10.1186/s12884-023-06238-5

**Published:** 2024-01-04

**Authors:** David Collins Agaba, Henry Mark Lugobe, Richard Migisha, Mark Jjuuko, Pascal Saturday, Dean Kisombo, Subira Mlangwa Atupele, Justus Kirabira, Matthew Tumusiime, Godfrey Katamba, Godfrey Mugyenyi, Sezalio Masembe, Musa Kayondo, Joseph Ngonzi

**Affiliations:** 1 Department of Physiology, Mbarara University of Science & Technology, P.O Box 1410, Mbarara, Southwestern Uganda; 2 Department of Obstetrics & Gynaecology, Mbarara University of Science & Technology, P.O Box 1410, Mbarara, Southwestern Uganda; 3 Department of Physiology, King Ceasor University, P.O Box 88, Kampala, Uganda; 4 Department of Obstetrics & Gynaecology, Kabale University, P.O Box 317, Kabale, Southwestern Uganda

**Keywords:** Obstetric shock index, Postpartum period, Prevalence, Pregnancy, Uganda

## Abstract

**Background:**

Early recognition of haemodynamic instability after birth and prompt interventions are necessary to reduce adverse maternal outcomes due to postpartum haemorrhage. Obstetric shock Index (OSI) has been recommended as a simple, accurate, reliable, and low-cost early diagnostic measure that identifies hemodynamically unstable women.

**Objectives:**

We determined the prevalence of abnormal obstetric shock index and associated factors among women in the immediate postpartum period following vaginal delivery at Mbarara Regional Referral Hospital (MRRH) in southwestern Uganda.

**Methods:**

We conducted a cross-sectional study at the labour suite and postnatal ward of MRRH from January 2022 to April 2022. We systematically sampled women who had delivered vaginally, and measured their blood pressures and pulse rates at 1 h postpartum. We excluded mothers with hypertensive disorders of pregnancy. Sociodemographic, medical and obstetric data were obtained through interviewer-administered questionnaires. The prevalence of abnormal OSI was the proportion of participants with an OSI ≥ 0.9 (calculated as the pulse rate divided by the systolic BP). Logistic regression analysis was used to determine associations between abnormal OSI and independent variables.

**Results:**

We enrolled 427 women with a mean age of 25.66 ± 5.30 years. Of these, 83 (19.44%), 95% CI (15.79–23.52) had an abnormal obstetric shock index. Being referred [aPR 1.94, 95% CI (1.31–2.88), *p* = 0.001], having had antepartum haemorrhage [aPR 2.63, 95% CI (1.26–5.73), *p* = 0.010] and having a visually estimated blood loss > 200 mls [aPR 1.59, 95% CI (1.08–2.33), *p* = 0.018] were significantly associated with abnormal OSI.

**Conclusion:**

Approximately one in every five women who delivered vaginally at MRRH during the study period had an abnormal OSI. We recommend that clinicians have a high index of suspicion for haemodynamic instability among women in the immediate postpartum period. Mothers who are referred in from other facilities, those that get antepartum haemorrhage and those with estimated blood loss > 200mls should be prioritized for close monitoring. It should be noted that the study was not powered to study the factors associated with AOSI and therefore the analysis for factors associated should be considered exploratory.

## Background

Obstetric shock index is a simple, low-cost early diagnostic tool used to identify women who are haemodynamically unstable due to acute blood loss that follows delivery [1, 2]. It is the ratio of heart rate to systolic blood pressure and has a normal range of 0.7-<0.9 [3, 4].

The obstetric shock index is more sensitive to hemodynamic changes that follow obstetric haemorrhage, compared to classic vital signs such as heart rate and systolic or diastolic blood pressure interpreted independently [5] or even visual estimation of blood loss which is inaccurate and underestimates the actual blood loss by up to 50% [6].

This is because postpartum haemorrhage results in a reduction in intravascular volume, venous return, cardiac out and mean arterial pressure that trigger an immediate compensatory increase in maternal heart rate due to baroreceptor reflex mediated sympathetic tone activation [7]. This is in a bid to keep the systolic blood pressure stable until no further increase in heart rate is possible, after which the systolic blood pressure begins to fall, usually after a woman has lost up to 30% of her blood volume [8].

Obstetric haemorrhage continues to be the leading cause of maternal mortality globally accounting for 27.1% of all maternal deaths [9] and 31% of all maternal deaths at Mbarara Regional Referral Hospital, southwestern Uganda [10]. In order to reduce adverse maternal outcomes due to haemorrhage [9, 11], early recognition of haemodynamic instability following haemorrhage and prompt intervention are necessary [12]. The obstetric shock index has been found to be a simple, accurate and reliable tool for early detection of hemodynamic instability following delivery [2, 5, 13] so that timely interventions can be instituted.

This study aimed to determine the prevalence of and factors associated with abnormal obstetric shock index among immediate postpartum women following vaginal delivery at Mbarara Regional Referral Hospital, a tertiary hospital in Uganda.

## Methods

### Study setting, study design and study population

This was a cross-sectional study conducted at the labour suite and post-natal ward of the department of Obstetrics and Gynaecology of Mbarara Regional Referral Hospital, southwestern Uganda from January 2022 to April 2022. Mbarara Regional Referral Hospital is one of the government-owned Regional Referral hospitals, located in South Western Uganda, about 260 km from the capital Kampala. The hospital has a catchment population of 12 districts and also serves as the teaching hospital for Mbarara University of Science and Technology (MUST). It has a bed capacity of about 350 beds 40 of which are on the postnatal ward and 3 beds in the labour suite.

The department conducts about 10,000 deliveries annually, about half of which are vaginal deliveries [14]. Following vaginal delivery, mothers are admitted to the post-natal ward for 24 h where they are assessed for haemodynamic stability using vital signs such as the blood pressure, pulse rate as well as their clinical state.

Women who had delivered vaginally at MRRH and were within the immediate postpartum period (first one hour after delivery of the placenta) were included into our study. We excluded patients admitted with any hypertensive disorder of pregnancy as hypertension would make interpretation of the shock index difficult since it would give a falsely normal shock index.

### Sample size and sampling

The sample size for the study was calculated using the Kish Leslie formula of 1965 for calculating sample size for cross-sectional studies [15]. Using 1.96 as the critical value at 5% level of significance, taking the prevalence of abnormal obstetric shock index among immediate postpartum women following vaginal delivery to be 50% since it was not known, 0.05 as a margin of error and adjusting for a nonresponse rate of 10%, we estimated a sample size of 427. Given that about 900 mothers deliver vaginally at MRRH within a period of 3 months, we used systematic sampling with a sampling interval of 2 (900/427) to recruit participants. Notably, we did not perform a specific sample size calculation for assessing factors associated with AOSI due to the absence of relevant reference studies on this topic. Written informed consent was obtained from each study participant before recruitment and participation in the study.

### Data collection and study variables

Data were collected by two research assistants using an interviewer-administered structured questionnaire. The research assistants were midwives, who were trained on the data collection tool and study procedures. Our outcome variable was obstetric shock index which was dichotomized as normal and abnormal obstetric shock index. We defined abnormal obstetric shock index as a ratio of pulse rate to systolic blood pressure ≥ 0.9 [4].

The questionnaire captured data on independent variables including socio-demographic, medical and obstetric factors. The variables included sociodemographic factors such as age, residence, employment status, religion, marital status, education level and referral status. Medical factors included BMI, HIV serostatus, systolic blood pressure, diastolic blood pressure and pulse rate. Obstetric factors included parity, gestational age at birth, birth weight, estimated blood loss, presence of perineal tears or episiotomies, augmentation/induction of labour, antepartum haemorrhage, multiple pregnancy and intrauterine foetal death. The variables that could not be ascertainable from the mothers such as visually estimated blood loss, use of uterotonics for induction or augmentation of labour were obtained from the participants’ charts.

Gestational age was calculated basing on the first day of last normal menstrual period. If a participant was unable to remember her LNMP, we estimated her gestation age using the first trimester ultrasound scan. Estimated blood loss was the blood loss documented in the file based on visual estimation of blood loss by the clinical team. A woman was considered to have been referred in from another health facility if she had a formal referral letter or any documentation of referral in her medical records. The HIV status was considered as the HIV status of the client in the 3 months preceding the interview as long as the result was documented. For women who did not have a documented result, we performed an HIV test on them. The research assistants examined the perineum for episiotomies and tears and a mother was considered to have had these if there was evidence of a laceration that had been sutured.

### Measurements

Blood pressure and pulse rate were measured at one hour after delivery of the placenta using an automated calibrated patient monitor (Mindray UMEC10 Vital Sign Patient Monitor, Shenzhen Mindray Bio-Medical Electronics Co., Ltd, China) to minimize user error and improve accuracy. An appropriate‑sized cuff that covered at least two-thirds of the length of the right upper arm and the entire circumference of the arm was attached as well as a pulsoximeter probe attached to the middle finger of the left arm. The participant was asked to remain quiet as the machine took measurements. The cuff inflated and deflated automatically after pressing the start button, and then displayed the BP and pulse rate on the screen of the monitor.

The weight of the participants was measured using a calibrated Secca weighing scale (Seca 762, GmbH & Co. KG, Hamburg, Germany) to the nearest second decimal. The participants had no shoes, nor heavy clothes on. The height was taken using a stadiometer to the nearest decimal in standing up position with the heel, buttock, and upper back along the same vertical plane.

### Study procedure

The first participant was identified using simple random sampling employing rotary method among the participants who had delivered vaginally and in the immediate postpartum period. If the sampled participant was not eligible for the study, the next immediate mother that had delivered vaginally was chosen and then resumed with the sampling interval of two for the next participant.

Once the participants were sampled, their blood pressures and pulse rates were measured at one hour after delivery. The obstetric shock index was then calculated and mothers with an index of 0.9 or more were linked to the clinical care team for further assessment and stabilisation before they could be recruited. Those with normal obstetric shock indices were immediately approached by the study team, assessed for eligibility, informed consent obtained and then recruited. Once consent had been obtained, the research team then administered the questionnaire and then measured the participants’ weight and height.

### Data management and analysis

Raw data were cross-checked for any discrepancies and completeness. The data were then double entered into Epi Data 3.1 (EpiData, Odense, Denmark) after which they were exported to STATA version 17 (StataCorp, College Station, Texas, USA) for cleaning and analysis. Independent variables were analysed using descriptive statistics. Continuous variables were described as mean ± SD while categorical variables were described as percentages. We used a chi-square test or Fisher’s exact test (in cases of small counts < 5) for categorical variables and a student t-test for continuous variables to compare baseline characteristics of participants with and without abnormal obstetric shock index.

The prevalence of abnormal obstetric shock index was calculated as the number of women with an obstetric shock index greater than or equal to 0.9 expressed as a percentage of the total participants.

To determine the factors associated with abnormal OSI, we used modified Poisson regression analysis; we used a generalized linear model with Poisson as family and a log link without an offset but including robust standard errors. Variables that had a *p*-value < 0.2 at bivariate analysis or were biologically plausible were entered into a multivariable modified Poisson regression model, step wise backwise elimination to identify factors independently associated (with *p* < 0.05) with abnormal OSI in the immediate postpartum period among women delivered vaginally at MRRH.

## Results

During the study period, 430 immediate postpartum mothers were screened, and 427 were ultimately included in the study. Three participants were excluded due to the presence of a hypertensive disorder of pregnancy, specifically severe pre-eclampsia.

### Socio-demographic and medical characteristics of participants

The mean age of the participants was 25.66 ± 5.30 years. The majority were married/lived with a partner (95.6%), Christian (92.97%), resided in urban areas (62.76%), HIV negative (90.4%) and overweight (51.05%) (Table 1). There was a higher proportion of referred mothers (49.4% vs. 27.03%, *p* < 0.001) among patients with abnormal Obstetric Shock index compared to those who had a normal obstetric shock index.

**Table 1:** Socio-demographic & medical characteristics of participants

Characteristic	Overall N = 427	AOSI n = 83	NOSI n = 344	***p***-value
	n (%)	n (%)	n (%)	
**Age in years (mean ± SD)**	25.66 ± 5.30	24.81 ± 4.60	25.86 ± 5.44	0.103
**Marital status (Married/Cohabiting)**				0.315
No	19 (04.55)	02 (10.53)	17 (89.47)	
Yes	408 (95.55)	81 (19.85)	327 (80.15)	
**Education Level**				0.653
Never attended	9 (2.11)	2 (22.22)	07 (77.78)	
≤Secondary	325 (76.11)	66 (20.31)	259 (79.69)	
Tertiary/University	93 (21.78)	15 (16.13)	78 (83.87)	
**Religion**				0.129
Moslem	30 (7.03)	9 (30)	21 (70)	
Christian	397 (92.97)	74 (18.64)	323 (81.36)	
**Employment status**				0.130
Unemployed	133 (31.15)	32 (24.06)	101 (75.94)	
Formal	88 (20.61)	19 (21.59)	69 (78.41)	
Informal	206 (48.24)	32 (15.53)	174 (84.47)	
**Area of residence**				0.630
Rural	159 (37.24)	29 (18.24)	130 (81.76)	
Urban	268 (62.76)	54 (20.15)	214 (79.85)	
**Referral Status**				**< 0.001**
No	293 (68.62)	42 (14.33)	251 (85.67)	
Yes	134 (31.38)	41 (30.60)	93 (69.40)	
**HIV Status**				0.414
Negative	386 (90.40)	77 (19.95)	309 (80.05)	
Positive	41 (9.60)	6 (14.63)	35 (85.37)	
**BMI categories**				0.783
Normal weight	110 (25.76)	23 (20.91)	87 (79.09)	
Overweight	218 (51.05)	43 (19.72)	175 (80.28)	
Obese	99 (23.19)	17 (17.17)	82 (82.83)	

***AOSI***: *Abnormal Obstetric Shock Index*   ***NOSI***: *Normal Obstetric Shock Index*  ***SD***: *Standard Deviation*

### Obstetric characteristics of participants

Of the 427 participants, majority had parity < 3 (64.17%), singleton pregnancies (97.19%) and were delivered at term (82.9%). The mean visually estimated blood loss at delivery was 201.64 ± 76.07 mls (Table 2). Compared to participants with Normal Obstetric Shock Index, those with Abnormal Obstetric shock Index had a higher mean visually estimated blood loss at delivery (243.01 ± 110.56 mls vs. 191.66 ± 61.24 mls, *p* < 0.0001), higher proportion of episiotomies/perineal lacerations (57.83% vs. 41.57%, *p* = 0.007) and higher proportions of APH (3.61% VS 0.29%, *p* = 0.024).

**Table 2:** Obstetric characteristics of participants

Characteristic	Overall N = 427	AOSI n = 83	NOSI n = 344	***p***-value
	n (%)	n (%)	n (%)	
**Parity**				0.213
<3	274 (64.17)	60 (21.90)	214 (78.10)	
3-<5	113 (26.46)	16 (14.16)	97 (85.84)	
≥5	40 (9.37)	7 (17.50)	33 (82.50)	
**Gestational age (WOA)**				0.236
< 37	56 (13.11)	7 (12.50)	49 (87.50))	
37–41	354 (82.90)	71 (20.06)	283 (79.94)	
>41	17 (3.98)	5(29.41)	12 (70.59)	
**Multiple pregnancy**				0.475
No	415 (97.19)	82 (19.76)	333 (80.24)	
Yes	12 (2.81)	01 (8.33)	11 (91.67)	
**Labour induced/augmented**				0.639
No	326 (76.35)	65 (19.94)	261 (80.06)	
Yes	101 (23.65)	18 (17.82)	83 (82.18)	
**Episiotomy/perineal tear**				**0.007**
No	236 (55.27)	35 (14.83)	201 (85.17)	
Yes	191 (44.73)	48 (25.13)	143 (74.87)	
**APH**				**0.024**
No	423 (99.06)	80 (18.91)	343 (81.09)	
Yes	04 (0.94)	03 (75)	01 (25)	
**Estimated blood loss**, mls, (mean ± SD)	201.64 ± 76.07	243.01 ± 110.56	191.66 ± 61.24	**< 0.0001**
**Estimated blood loss**				**0.014**
≤ 200mls	304 (71.19)	50 (16.45)	254 (83.55)	
>200mls	123 (28.81)	33 (26.83)	90 (73.17)	
**Birth weight (kg)**				0.233
< 3.5	358 (83.84)	66 (18.44)	292 (81.56)	
≥ 3.5	69 (16.16)	17 (24.64)	52 (75.36)	

***WOA***: *Weeks of Amenorrhea*;  ***BMI***: *Body Mass Index*;  ***NOSI***: *Normal Obstetric Shock Index*;  ***AOSI***: *Abnormal Obstetric Shock Index*;  ***SD***: *Standard Deviation*;  ***SBP***: *Systolic blood pressure*;  ***DBP***: *Diastolic Blood Pressure*;  ***PR***: *Pulse Rate*

### Prevalence of abnormal obstetric shock index

Out of the 427 participants recruited, 83 had an Abnormal Obstetric Shock Index giving the prevalence of Abnormal Obstetric Shock Index among immediate postpartum women following vaginal delivery at MRRH of 19.44% (95%CI 15.79–23.52).

### Factors associated with abnormal obstetric shock index

At multivariable analysis, being referred [aPR 1.94, 95% CI (1.31–2.88), *p* = 0.001], having had antepartum haemorrhage [aPR 2.63, 95% CI (1.26–5.47), *p* = 0.010] and having had a visually estimated blood loss > 200 mls [aPR 1.59, 95% CI (1.08–2.33), *p* = 0.018] were independently associated with abnormal obstetric shock index (Table 3).

**Table 3:** Factors associated with abnormal obstetric shock index at multivariable modified poisson regression

Characteristic	% AOSI n/N (%)	Bivariate analysis	***p***-value	Multivariable analysis	***p***-Value	
		cPR (95% CI)		aPR (95% CI)		
**Age (years)**						
<25	46 (22.44)	Ref		Ref		
25-<35	32 (16.93)	0.75 (0.50–1.13)	0.174	0.96 (0.57–1.63)		0.893
≥35	05 (15.5)	0.68 (0.29–1.58)	0.364	1.10 (0.43–2.83)		0.837
**Employment status**						
Employed	51(17.35)	Ref		Ref		
Unemployed	32(24.06)	1.39 (0.94–2.05)	0.102	1.270 (0.83–1.95)		0.263
**Referral Status**						
No	42(14.33)	Ref		Ref		
Yes	41(30.60)	**2.13 (1.46–3.12)**	**< 0.001**	**1.94 (1.31–2.88)**		**0.001**
**Parity**						
<3	60(21.90)	Ref		Ref		
3-<5	16(14.16)	0.65 (0.39–1.07)	0.092	0.80 (0.43–1.48)		0.474
≥5	07(17.50)	0.80 (0.39–1.63)	0.536	0.96 (0.41–2.27)		0.933
**Gestational age (WOA)**						
< 37	07(12.50)	0.62 (0.30–1.29)	0.201	0.62 (0.30–1.29)		0.201
37–41	71(20.06)	Ref		Ref		
>41	05(29.41)	1.46 (0.68–3.15)	0.327	1.46 (0.76–2.81)		0.258
**Multiple pregnancy**						
No	82 (19.76)	Ref		Ref		
Yes	01 (8.33)	0.42 (0.06–2.79)	0.370	0.423 (0.07–2.41)		0.330
**Episiotomy/laceration**						
No	35(14.83)	Ref		Ref		
Yes	48(25.13)	1.69 (1.14–2.51)	0.008	1.44 (0.91–2.28)		0.120
**APH**						
No	80 (18.91)	Ref		Ref		
Yes	03 (75)	**3.97 (2.18–7.22)**	**< 0.001**	**2.63 (1.26–5.47)**		**0.010**
**Blood loss (mls)**						
≤ 200	50 (16.45)	Ref		Ref		
>200	33 (26.83)	**1.63 (1.11–2.40)**	**0.013**	**1.59 (1.08–2.33)**		**0.018**

**cPR**: crude Prevalence Ratio; **aPR**: Adjusted Prevalence Ratio; **Ref**: Reference group; **CI**: Confidence Interval; **APH**: Antepartum Haemorrhage

## Discussion

Following haemorrhage, an abnormal obstetric shock index is an early marker of haemodynamic instability because the cardiovascular system adjustments that follow PPH cause an early elevation of the heart rate before the blood pressure begins to fall [16]. This study determined the prevalence of and factors associated with abnormal obstetric shock index among women in the immediate post-partum period following vaginal delivery at a tertiary hospital in southwestern Uganda. About one in every 5 women who delivered vaginally at MRRH, had an abnormal obstetric shock index one hour after delivery and women who had been referred, had antepartum haemorrhage and those who had visually estimated blood loss after delivery > 200mls were more likely to have an abnormal obstetric shock index.

There is a paucity of literature on the prevalence of abnormal obstetric shock index in the immediate postpartum period making it difficult to compare our findings. However, this prevalence is comparable to the proportion among women with no PPH (16%) in a retrospective case-control study done at St. George’s hospital London to determine the usefulness of the “obstetric shock index” as an adjunct in identifying significant blood loss in mothers with massive postpartum hemorrhage [3]. This is probably because this proportion was among women who had no PPH just like in our population where the majority of the patients had no PPH (99.77%). However, the prevalence in the current study is lower than the proportions of women who had abnormal obstetric shock indices in cohort studies done in South Africa (45.9%) [17] and the UK (58.8%) [18]. The above studies had much higher proportions because they only enrolled women with PPH, a population that is more likely to have haemodynamic instability. Additionally, where as we recruited only women who had delivered vaginally, these studies recruited women who delivered vaginally as well as by caesarean section and yet caesarean delivery is associated with higher volumes of blood loss.

In our study, women who were referred were more likely to have an abnormal obstetric shock index in the immediate postpartum period compared to those who were not referred. In our setting, women are referred intrapartum due to complications of labour such as antepartum haemorrhage (APH), prolonged labour, and suffer complications such as dehydration and postpartum haemorrhage [19]. This morbidity is further compounded by the delays that affect health care systems in low- and middle-income countries such lack of readily available transport, poor roads, bad geographical terrain, long distances to the referral facilities, stock outs of medicines and supplies [20]. As a result, the majority of the women reach the referral facilities when the complications have worsened and therefore at risk of postpartum haemorrhage and therefore abnormal obstetric shock index. There is therefore a need to improve our referral systems through timely referrals and addressing the delays that affect the referral system.

Women who had had antepartum haemorrhage were more likely to have an abnormal obstetric shock index compared to those who had not. Hemorrhage leads to a decrease in intravascular volume, subsequently reducing venous return, cardiac output, and mean arterial pressure. Consequently, there is a compensatory rise in maternal heart rate, driven by baroreceptor reflex-mediated sympathetic activation, aimed at maintaining normal blood pressure. These findings underscore the need for a thorough assessment and timely resuscitation of women experiencing antepartum hemorrhage.

Participants who had a visually estimated blood loss > 200 mls were more likely to have an AOSI compared to those who had blood loss ≤ 200 mls. This volume threshold is much lower than the standard 500mls defined by WHO as the volume that is used to define postpartum haemorrhage (PPH) & haemodynamic instability following vaginal delivery [21]. This low threshold could have been due to underestimation of blood loss given that visual estimation of blood loss, the method we used has been shown to underestimate blood loss by up to 50% [6]. To note, in our study, only one patient had an estimated blood loss in the PPH range (≥ 500mls).

Our study was not without limitations. The study relied on the visually-estimated blood loss recorded in the patient’s file, a method known to underestimate blood loss by up to 50%. This may potentially bias our observed associations towards the null. However, all deliveries were conducted by qualified birth attendants, ensuring a consistent approach for blood loss estimation. Additionally, we recognize that we may have been underpowered to comprehensively study factors associated with AOSI, as we did not conduct a specific sample size calculation for this objective. Due to a lack of pre-planned power calculation, the analysis of factors associated with AOSI was exploratory in nature. Future longitudinal studies with pre-planned sample sizes for risk factor analyses would provide more robust evidence on the risk factors of AOSI in our setting.

## Conclusions

The study found that about 1 in every 5 women delivered vaginally at Mbarara Regional Referral hospital southwestern Uganda had an abnormal obstetric shock index in the immediate postpartum period. Women who were referred, those who had had antepartum haemorrhage and those with an estimated blood loss > 200mls were more likely to have an abnormal obstetric shock index compared to their counterparts. We recommend that clinicians have a high index of suspicion for haemodynamic instability among women in the immediate postpartum period following vaginal delivery. Additionally, women who have been referred, those who have had antepartum haemorrhage and those with estimated blood loss > 200 mls should be prioritized for close monitoring in the immediate postpartum period following vaginal delivery.

## Data Availability

The data and materials used to support the findings of this study are available on request from the corresponding author.

## Abbreviations

APH: Antepartum Haemorrhage
aOR: adjusted Odds Ratio
AOSI: Abnormal Obstetric Shock Index
BMI: Body Mass Index
BP: Blood Pressure
DBP: Diastolic Blood Pressure
CI: Confidence Interval
cOR: Crude Odds Ratio
HTN: Hypertension
ICU: Intensive Care Unit
Mls: Milliliters
MRRH: Mbarara Regional Referral Hospital
MUST: Mbarara University of Science and Technology
NOSI: Normal Obstetric Shock Index
OPD: Out Patients Department
OSI: Obstetric Shock Index
PPH: Postpartum Haemorrhage
PR: Pulse Rate
Ref: Reference group
SBP: Systolic Blood Pressure
SD: Standard Deviation
SI: Shock Index
UNCST: Uganda National Council of Science and Technology
WHO: World Health Organization
